# Three-dimensional finite element analysis of stress distribution on short implants with different bone conditions and osseointegration rates

**DOI:** 10.1186/s12903-023-02945-9

**Published:** 2023-04-15

**Authors:** Yunhe Yang, Yuchen Liu, Xi Yuan, Mingfa Ren, Xiaodong Chen, Lailong Luo, Lang Zheng, Yang Liu

**Affiliations:** 1 Graduate School of Dalian Medical University, Dalian, China; 2 Department of Engineering Mechanics, Dalian University of Technology, Dalian, China; 3 Graduate School of Dalian University, Dalian, China; 4 State Key Laboratory of Structural Analysis for Industrial Equipment, Dalian University of Technology, Dalian, China; 5 Department of Prosthodontics, Dalian Stomatological Hospital, Dalian, 116021 China

**Keywords:** Anisotropy, Bone condition, Finite element analysis, Osseointegration rate, Short implants

## Abstract

**Objective:**

This experiment aimed to investigate the effects of bone conditions and osseointegration rates on the stress distribution of short implants using finite element analysis and also to provide some reference for the application of short implants from a biomechanical prospect.

**Materials and methods:**

Anisotropic jaw bone models with three bone conditions and 4.1 × 6 mm implant models were created, and four osseointegration rates were simulated. Stress and strain for the implants and jaws were calculated during vertical or oblique loading.

**Results:**

The cortical bone area around the implant neck was most stressed. The maximum von Mises stress in cortical bone increased with bone deterioration and osseointegration rate, with maximum values of 144.32 MPa and 203.94 MPa for vertical and inclined loading, respectively. The osseointegration rate had the greatest effect on the maximum principal stress in cortical bone of type III bone, with its value increasing by 63.8% at a 100% osseointegration rate versus a 25% osseointegration rate. The maximum and minimum principal stresses under inclined load are 1.3 ~ 1.7 and 1.4 ~ 1.8 times, respectively, those under vertical load. The stress on the jaw bone did not exceed the threshold when the osseointegration rate was ≥ 50% for Type II and 100% for Type III. High strain zones are found in cancellous bone, and the maximum strain increases as the bone condition deteriorate and the rate of osseointegration decreases.

**Conclusions:**

The maximum stress in the jaw bone increases as the bone condition deteriorates and the osseointegration rate increases. Increased osseointegration rate reduces cancellous bone strain and improves implant stability without exceeding the yield strength of the cortical bone. When the bone condition is good, and the osseointegration ratio is relatively high, 6 mm short implants can be used. In clinical practice, incline loading is an unfavorable loading condition, and axial loading should be used as much as possible.

## Introduction

Implants have been widely used in the repair of dentition defects and loss in recent years due to long-term sound clinical results and have become an important means of restoring masticatory function, stabilizing occlusion, and improving aesthetics and pronunciation [1]. However, in case of limited anatomical conditions or bone resorption, the residual vertical bone of the jaw no longer becomes sufficient to support standard-length implants. Thus short or ultrashort implants have been introduced as alternatives to conventional implants [2, 3]. In 2016, the European consensus defined short implants as ≤ 8 mm and ultra-short implants as < 6 mm at the meeting [4]. A survey revealed that short implants were not significantly different from conventional implants in implant survival and bone stability when used to repair severely atrophied maxillae and mandibles [5].

Successful implants have a favorable biomechanical environment in which the implants function by transferring and dispersing occlusal loads to neighboring tissues. Occlusal overload concentrates the stress in the bone around the implant, causing bone resorption and even implant shedding. Different in vitro methods have been used for investigating the effect of loads on implants and their surrounding bones, such as photoelastic resins, digital image correlation (DIC), strain gauges, and three-dimensional finite element analysis [6, 7]. Presently, the three-dimensional finite element analysis is the most widely used numerical program for studying such problems since it is capable of recreating the mechanical behavior of materials under loading based on known properties, with the characteristics of high simulation and accurate calculation [8].

To assess the stress distribution of custom anatomical root implants at the bone implant interface under different bone conditions, Pawhat Nimmawitt et al. modeled four types of jaw bone with reference to Lekholm and Zarb's bone classification method [9]. Alexander Tsouknidas et al. developed an isotropic jaw model representing four types of jaws in an experiment to assess the effect of different bone conditions on peri-implant bone stress distribution, but accurate modeling of bone is a challenge due to its inherent inhomogeneity and anisotropic features [10]. Most past studies have assumed that bone is isotropic, and this simplification resulted in much lower stress predictions in peri-implant bone than in reality [11]. Therefore, establishing an anisotropic jaw model plays an active role in maintaining the authenticity of finite element analysis.

Sound osseointegration is a marker of long-term success in implant therapy and a basis for carrying various loads. Presently, the implant-bone osseointegration rate is generally assumed to be 100%; however, this is not consistent with clinical practice [12]. Roberts et al. discovered less than 50% osseointegration of clinically successful implants; Barbier et al. approximately reported implant-bone osseointegration rates to be 30%–70% [13, 14]. Thus, in recent years, some scholars have given more attention to the effects of different osseointegration rates on stress distribution. Duygu Yazicioglu et al. used three-dimensional finite element analysis to assess the stress distribution in short implants at various osseointegration rates and developed a model with a 70% osseointegration rate deemed more credible than the previously used complete osseointegration model [15]. Tetsuo Ohyama et al. obtained a high osseointegration rate of 98.2% by "photofunctionalizing" the titanium implant. They investigated stress distribution at osseointegration rates of 98.2% and 53.0%. This demonstrates the importance of studying the impact of incomplete osseointegration between the implant and its surrounding bone on biomechanical properties from a practical point of view [16].

The maximum von Mises stress, maximum principal stress, minimum principal stress, maximum shear stress, and maximum strain of the bone around short implants were obtained for different bone conditions and osseointegration rates under vertical and inclined loading in this study to provide some reference and experimental basis for the clinical application of short implants from a biomechanical standpoint. The null hypothesis states that bone condition and osseointegration rate do not affect stress distribution in the mandibular posterior region when 6 mm short implants are used.

## Materials and methods

### Experimental groups

The stress distribution characteristics of short implants with different bone conditions and osseointegration rates when vertically or obliquely loaded were investigated in this experiment. Cortical bone thickness was adjusted according to the bone classification method proposed by Lekholm and Zarb. In order to create three jaw models with different bone conditions: II, III, and IV, cancellous bone was divided into two types: high density and low density. 4.1 mm × 6 mm ITI standard soft tissue-level implants were simulated with a round superior abutment (5 mm in height). A transition region was introduced at the implant-bone interface to establish different osseointegration rates of 25%, 50%, 75%, and 100%, respectively, and a total of 24 groups were set up as described above.

### Model development

#### Establishment of mandible models

**Fig. 1:**
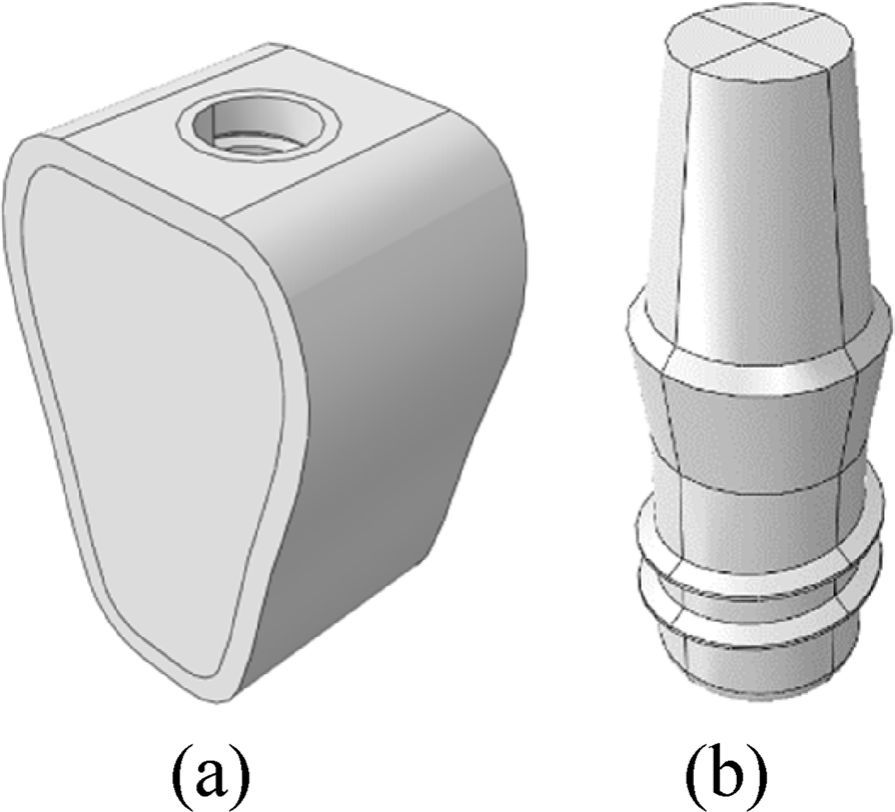
**a** Mandible model, **b** Implant model

As shown in Fig. 1, the mandible was developed using Solidworks 2019 three-dimensional modeling software. Around the implant neck, measurements of 20 mm jaw height, 15 mm maximum buccolingual width, and 10 mm buccolingual and mesiodistal width were taken. The models were generated with reference to Lekholm and Zarb’s classification principle of type II, III, and IV bones: the type II cortical bone is thicker, type III and type IV cortical bones are thinner, and type IV cancellous bones are less dense. Table 1 shows the three bone condition characteristics.

**Table 1:** Bone characteristics of type II, III, and IV bones

Bone types	Cortical bone thickness	Cancellous bone density
II	2 mm	High density
III	1 mm	High density
IV	1 mm	Low density

#### Establishment of implant models

As shown in Fig. 1b, Solidworks 2019 3D modeling software was adopted for drawing to simulate ITI regular columnar cervical soft tissue-level implants (Straumann, Switzerland). The diameter of the implants was 4.1 mm, the length was 6 mm, the thread spacing was 1.25 mm, the thread depth was 0.35 mm, the thread angle was 15°, the smooth neck height was 2.8 mm, and the neck diameter was 4.8 mm. The superior abutment at the height of 5 mm was simulated, and the abutment and implant were simplified into a single unit.

### Material properties and boundary constraints

#### Material properties of jaw bones

**Fig. 2:**
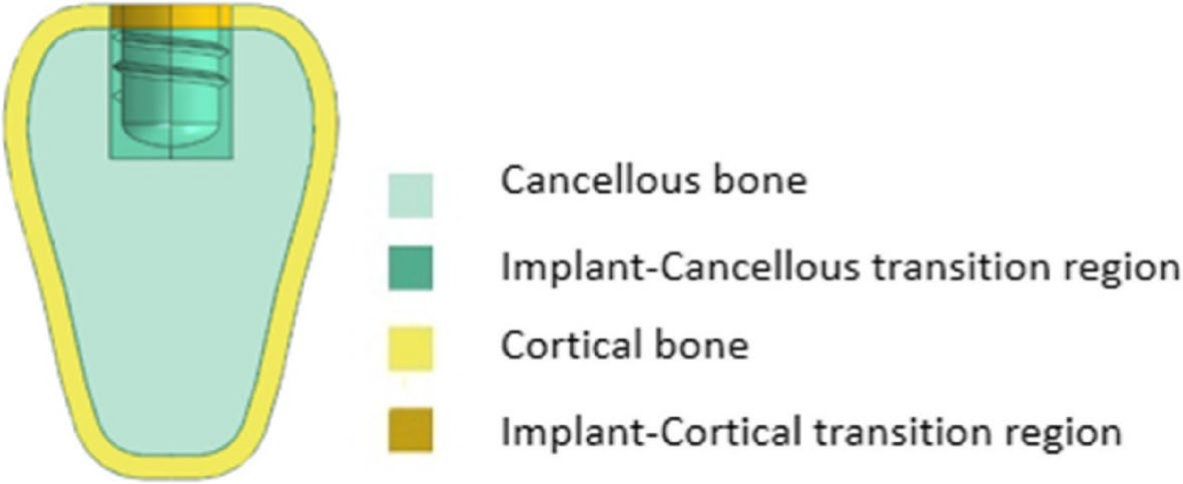
Regional division of mandible

As shown in Fig. 2, the cortical bone, cancellous bone, and implant-bone transition regions were divided in the mandible model. Emphasis is laid on the simulation of the implant-bone transition region, where a transition zone is introduced. The transition region is the portion of the jaw adjacent to the implant that lies outside the implant geometry and is the portion 0.5 mm away from the inner diameter of the implant. Different osseointegration rates were simulated by decreasing the material properties in this region.

Type II, III, and IV bones presented in this study are continuous elastic and anisotropic. Table 2 shows the parameters derived from a related study by Professors Williams and O'Mahony for high-density cancellous bone, low-density cancellous bone, and cortical bone [17, 18]. Where E is Young’s modulus, G is shear modulus, and *v* is Poisson’s ratio. These parameter values were used to realize the overall properties of cortical and cancellous bone and the transition zones with different rates of osseointegration [19].

**Table 2:** Material properties of the jaw bones

Materials	BIC	*E*_*x*_*(MPa)*	*E*_*y*_*(MPa)*	*E*_*z*_*(MPa)*	*v*_*xy*_	*v*_*xz*_	*v*_*yz*_	*G*_*xy*_*(MPa)*	*G*_*xz*_*(MPa)*	*G*_*yz*_*(MPa)*
High-density cancellous bone	25%	287	52.5	287	0.05	0.32	0.01	17	108.5	17
	50%	574	105	574				34	217	34
	75%	861	158	861				51	325.5	51
	100%	1148	210	1148				68	434	68
Low-density cancellous bone	25%	57.5	10.5	57.5	0.05	0.32	0.01	3.5	21.75	3.5
	50%	115	21	115				7	43.5	7
	75%	172.5	31.5	172.5				10.5	65.25	10.5
	100%	230	42	230				14	87	14
Cortical bone	25%	3150	3150	4850	0.3	0.253	0.25	1212.5	1425	1425
	50%	6300	6300	9700				2425	2850	2850
	75%	9450	9450	14550				3637.5	4275	4275
	100%	12600	12600	19400				4850	5700	5700

#### Material properties of implants

Titanium implants (and abutments) are presumed to be homogeneous and isotropic linear elastic materials. Elastic modulus E = 110 GPa, and Poisson’s ratio *v* = 0.35.

### Loads and boundary conditions

1. Vertical loading: The perpendicular static concentrated loading in the center of the abutment, which was 200N.
2. Inclined loading: The static concentrated loading on the buccal aspect at an angle of 45° to the long axis of the implant, which was 100N.

**Fig. 3:**
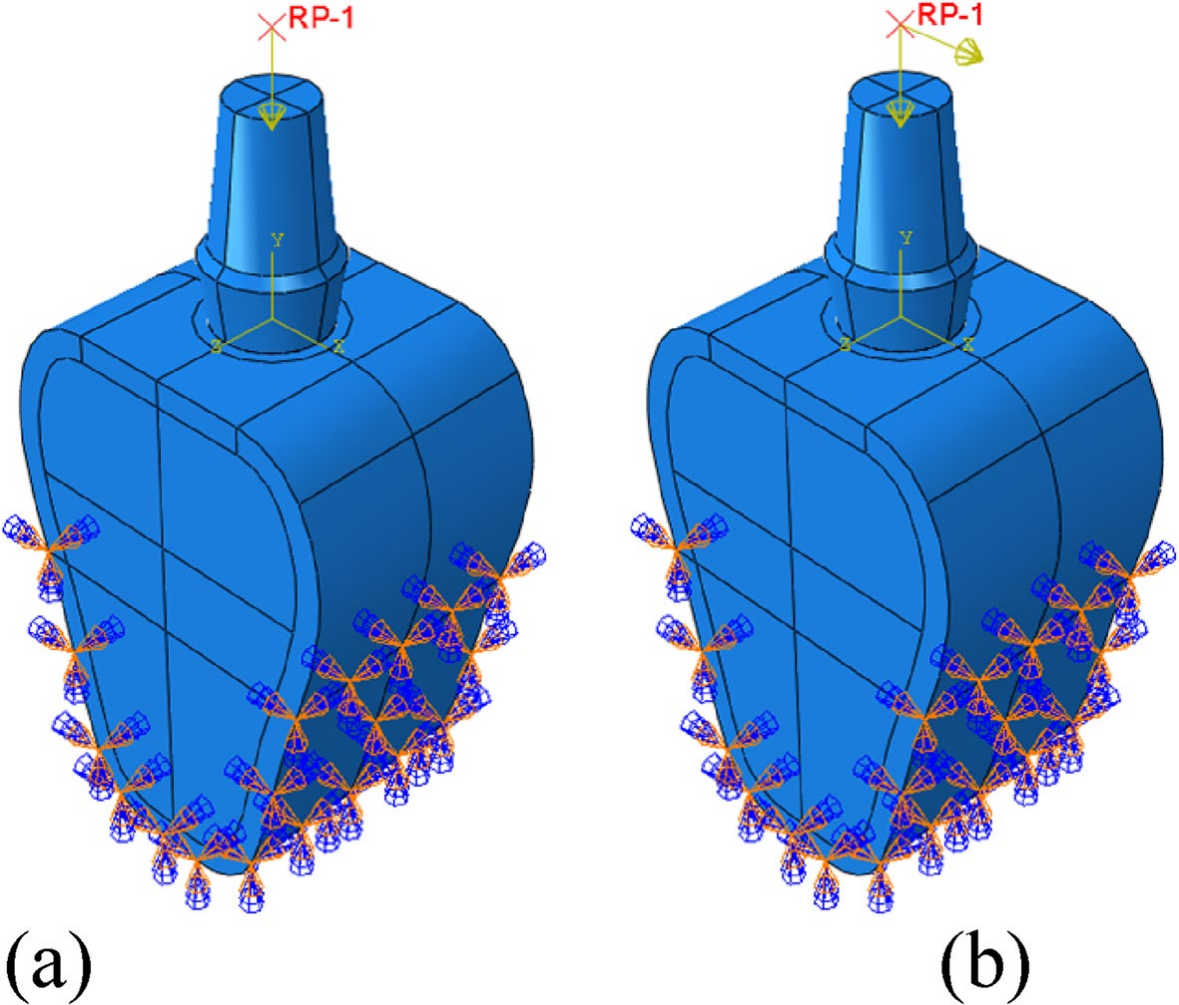
Schematic diagram of boundary conditions and loads. **a** Vertical loading, **b** Inclined loading

The abutment surface was coupled to the reference point, and loads were applied to the same. The implant-bone interface was bound, and full fixation constraints were imposed on the buccolingual and inferior surfaces of the bone. Figure 3 shows the loads and boundary conditions.

### Meshing and finite element analysis

**Fig. 4:**
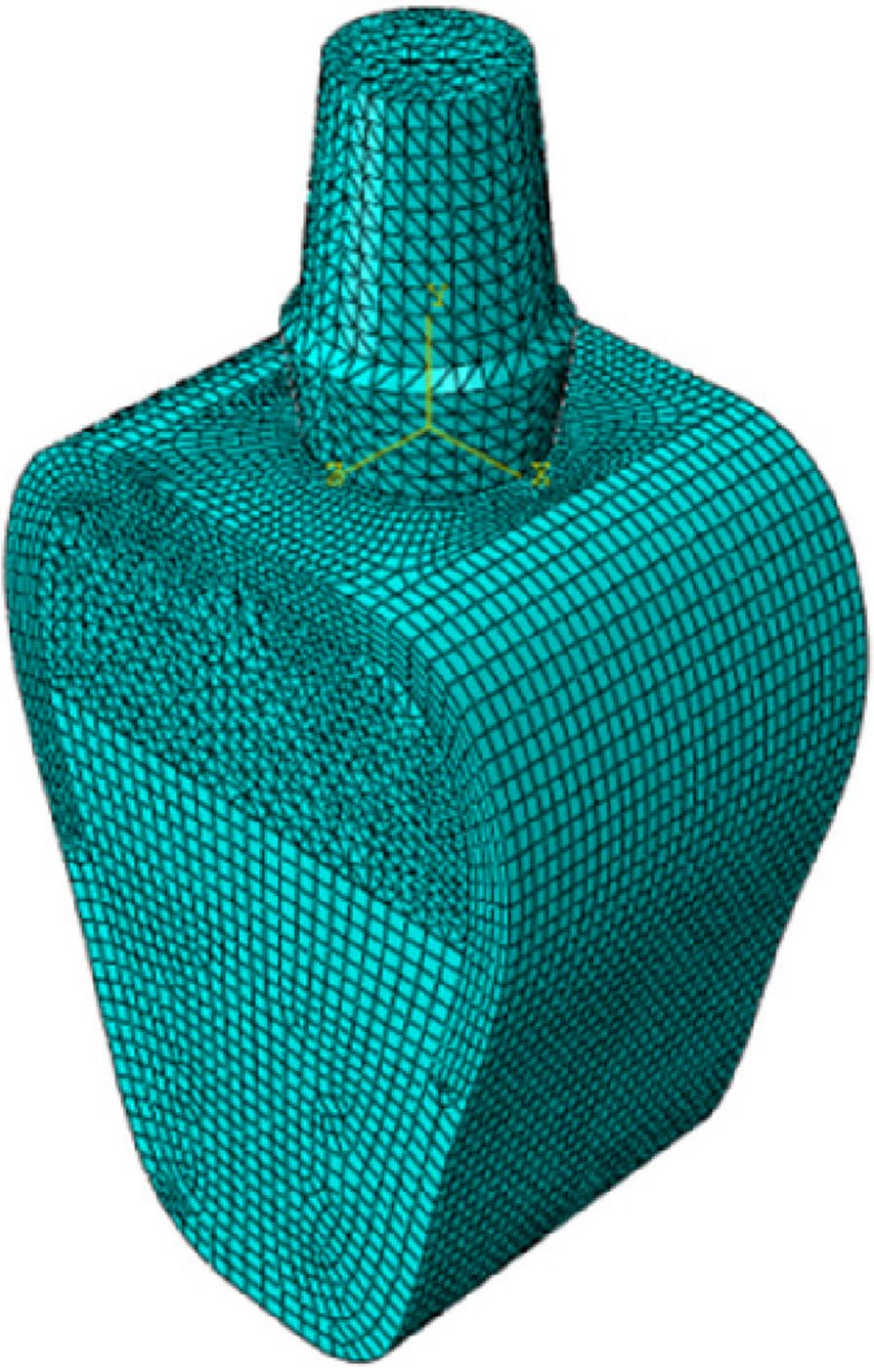
Schematic diagram of meshing

The mesh was completely refined until the difference between the model's maximum von Mises stress was < 1%. Based on the assumption of ensuring calculation accuracy, increasing calculation speed, and determining the mesh size as 0.5 mm for the implant, 0.4 mm for the cortical and cancellous bone, and 0.2 mm for the bone union transition area. To improve calculation accuracy, the hexahedral element (C3D8R) is preferred, which includes cortical bone, the lower part of cancellous bone, and the transition region. Tetrahedral elements (C3D10) are used in areas where hexahedral elements cannot be divided easily, such as the upper part of cancellous bone and implants. Figure 4 shows the meshing, and Table 3 shows the number of nodes and elements between the mandible and the implant.

**Table 3:** Nodes and elements of the finite element models

Materials	Nodes	Elements
Bone model	165781	118095
Implant	24652	16678

For static, general calculations, use abaqus 2020 finite element software. The maximum von mises stress, maximum strain, maximum principal stress, minimum principal stress, and maximum shear stress of the implant-bone junction were analyzed and compared under various bone conditions and bone integration rates.

## Results

### Maximum von mises stress & maximum strain

**Fig. 5:**
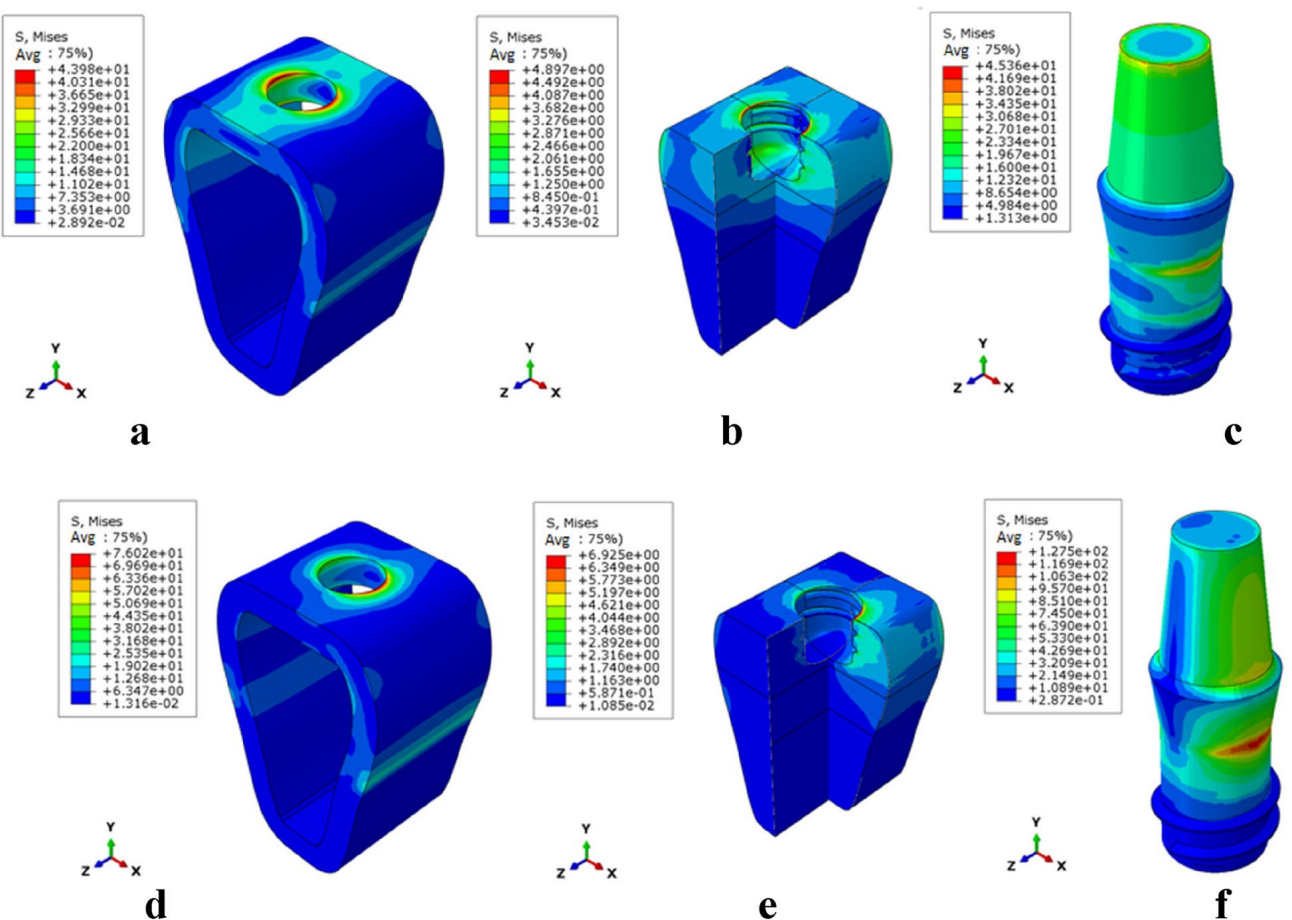
Von Mises stress distribution in Type II bone at 100% osseointegration. Under axial loading: **a** cortical bone, **b** cancellous bone, **c** implant. Under oblique loading: **d** cortical bone, **e** cancellous bone, and **f** implant

**Fig. 6:**
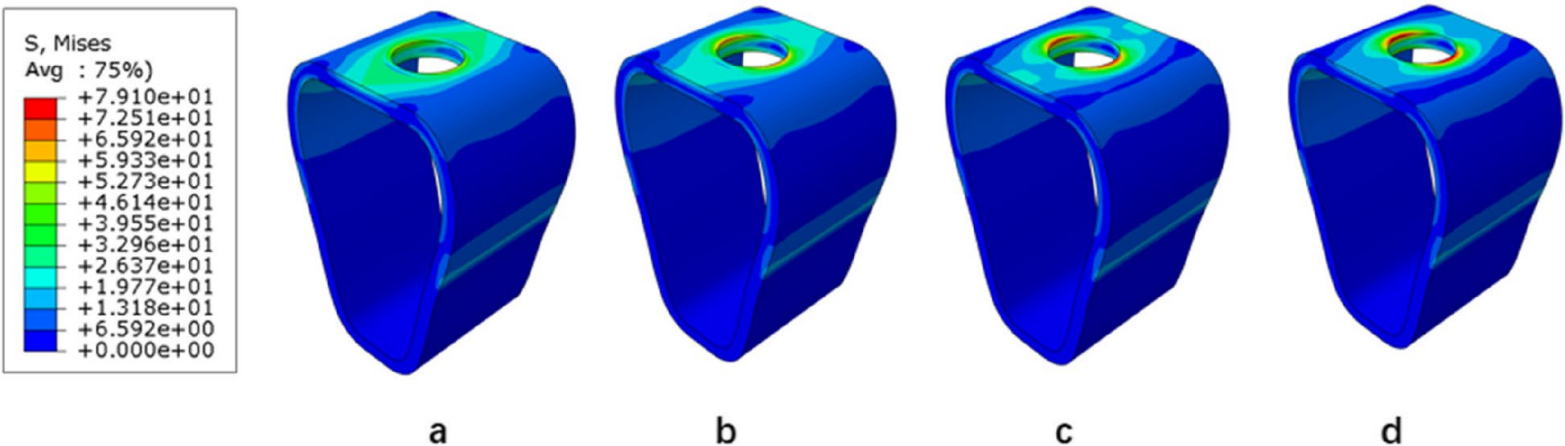
Maximal von mises stress in cortical bone (Type II) under vertical loading. **a** 25%, **b** 50%, **c** 75%, and **d** 100%

**Fig. 7:**
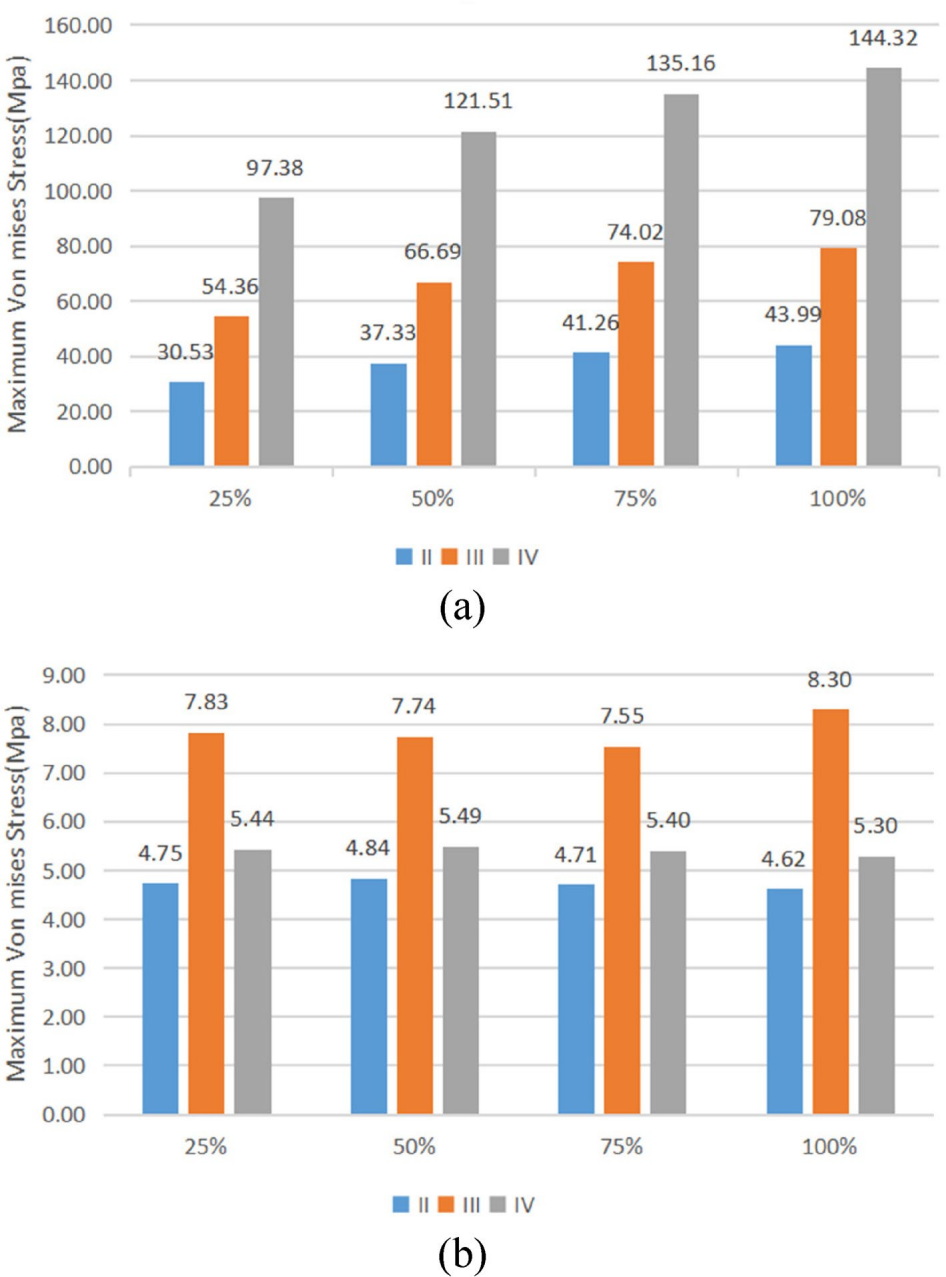
Maximal von mises stress versus bone conditions at the same osseointegration rate. **a** cortical bone, **b** cancellous bone

**Fig. 8:**
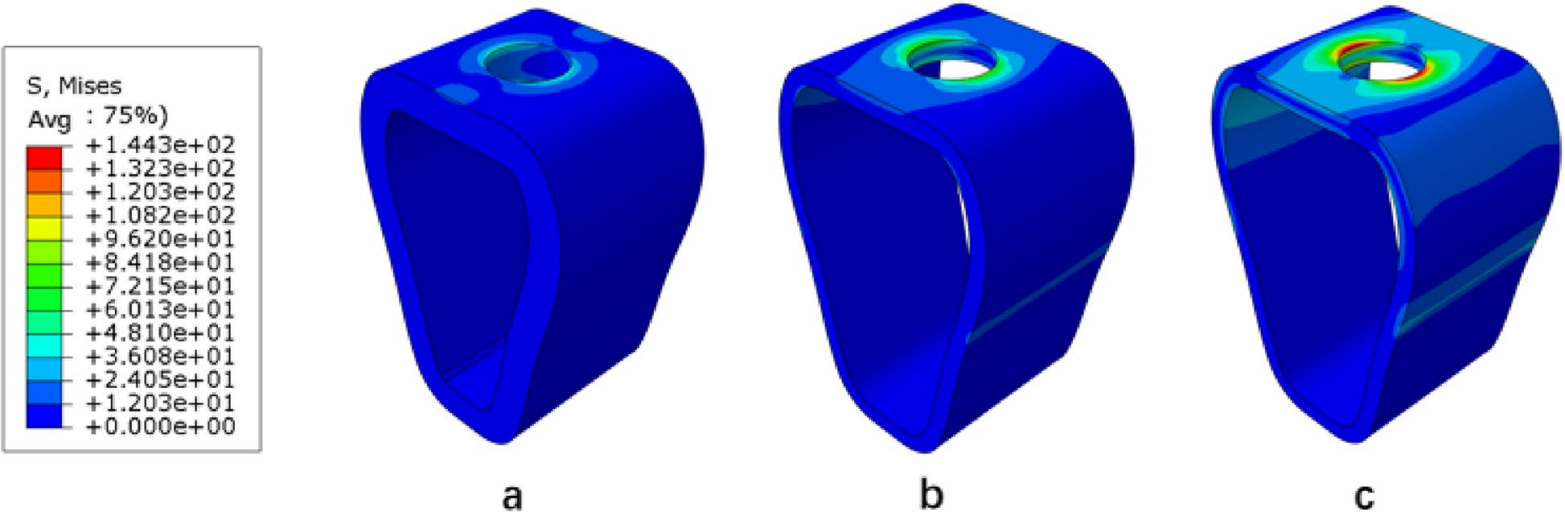
Maximum von mises stress in the cortical bone at a 100% osseointegration rate under axial loading. **a** Type II (**b**), Type III (**c**), and Type IV

The stress distribution patterns of cortical bone, cancellous bone, and implant were similar for different bone types and osseointegration rates. The maximum von mises stress was concentrated in the neck of the cortical bone close to the implant for the jaw bones, whereas the stress in cancellous bone was small and relatively uniform; in the case of the implant, the maximum von mises stress was located at the implant-cortical bone interface (Fig. 5). With the same bone type, cortical bone, and implant stress increased as osseointegration rates increased, with the maximum stress occurring when the osseointegration rate reached 100% (Fig. 6). Table 4 displays the specific values. With the same osseointegration rate, the maximum von mises stress in cortical bone in descending order is given as Type IV bone > Type III bone > Type II bone (Figs. 7a and 8). In contrast, for cancellous bone, the maximum von mises stress in descending order was Type III bone > Type IV bone > Type II bone (Fig. 7b). The stress value under inclined loading was greater than that under vertical loading. For the cortical bone, the maximum Von mises stress value under oblique loading was nearly 1.4 ~ 1.8 times higher than that under axial loading; for the implant, the corresponding value was 1.8 ~ 3.5 times higher. Moreover, it was found that when the bone condition was worse, the difference between the two values was smaller; for instance, the maximum difference was 3.1 ~ 3.5 times for Type II bone and 1.8 ~ 2.1 times for Type IV bone (Table 4).

**Table 4:** Maximum von mises stress values

Bone type		II				III				IV			
BIC		25%	50%	75%	100%	25%	50%	75%	100%	25%	50%	75%	100%
Vertical load	Cortical (MPa)	30.53	37.33	41.26	43.99	54.36	66.69	74.02	79.08	97.38	121.51	135.16	144.32
	Cancellous (MPa)	4.75	4.84	4.71	4.62	7.83	7.74	7.55	8.30	5.44	5.49	5.40	5.30
	Implant (MPa)	35.73	38.02	40.62	42.44	49.98	57.18	61.50	64.48	81.53	96.15	104.26	109.59
Lateral load	Cortical (MPa)	54.92	64.81	71.22	76.05	91.80	111.24	123.18	131.62	144.45	174.42	191.92	203.94
	Cancellous (MPa)	7.88	6.52	6.03	6.41	11.86	11.21	11.62	12.72	7.04	6.35	5.93	5.64
	Implant (MPa)	123.44	126.92	129.44	131.69	139.92	148.04	153.50	157.60	166.78	180.81	189.48	195.64

**Fig. 9:**
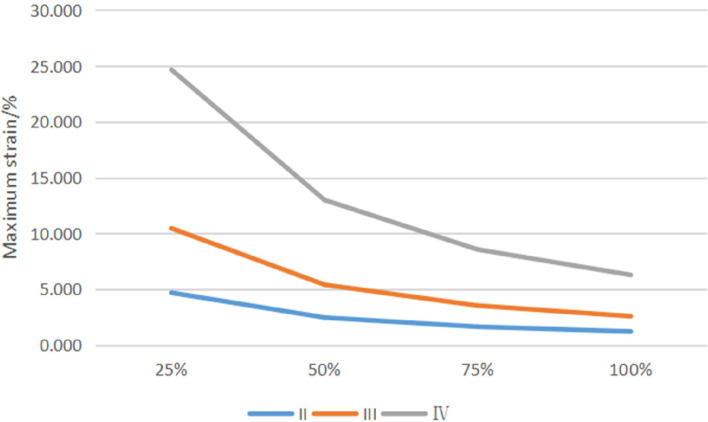
Maximum strain of cancellous bone

The cancellous bone area around the implant threads experiences the most strain. The strain size decreases as osseointegration improves and increases as bone conditions deteriorate (Fig. 9).

### Maximum and minimum principal stress

**Fig. 10:**
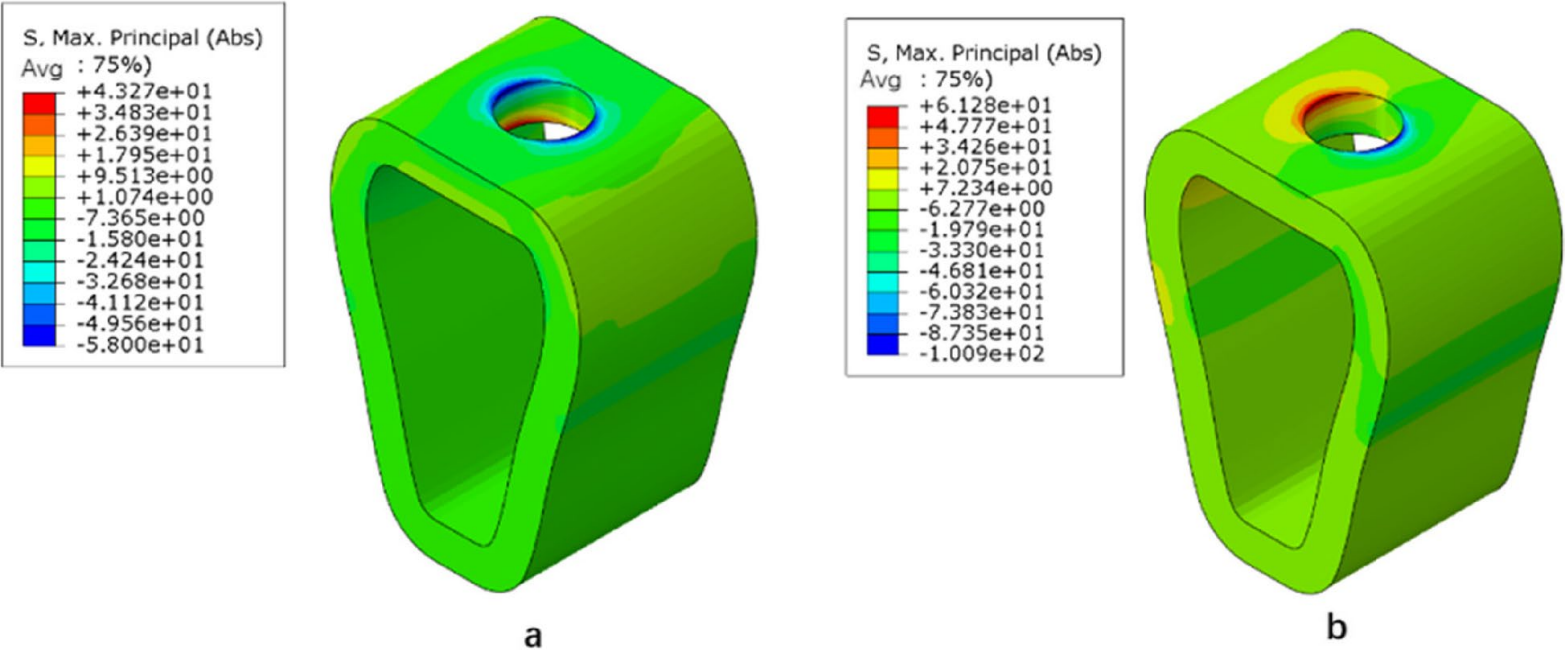
Type II bone, distribution plot of maximum/minimum principal stresses at 100% osseointegration. **a** axial loading (**b**) oblique loading

**Fig. 11:**
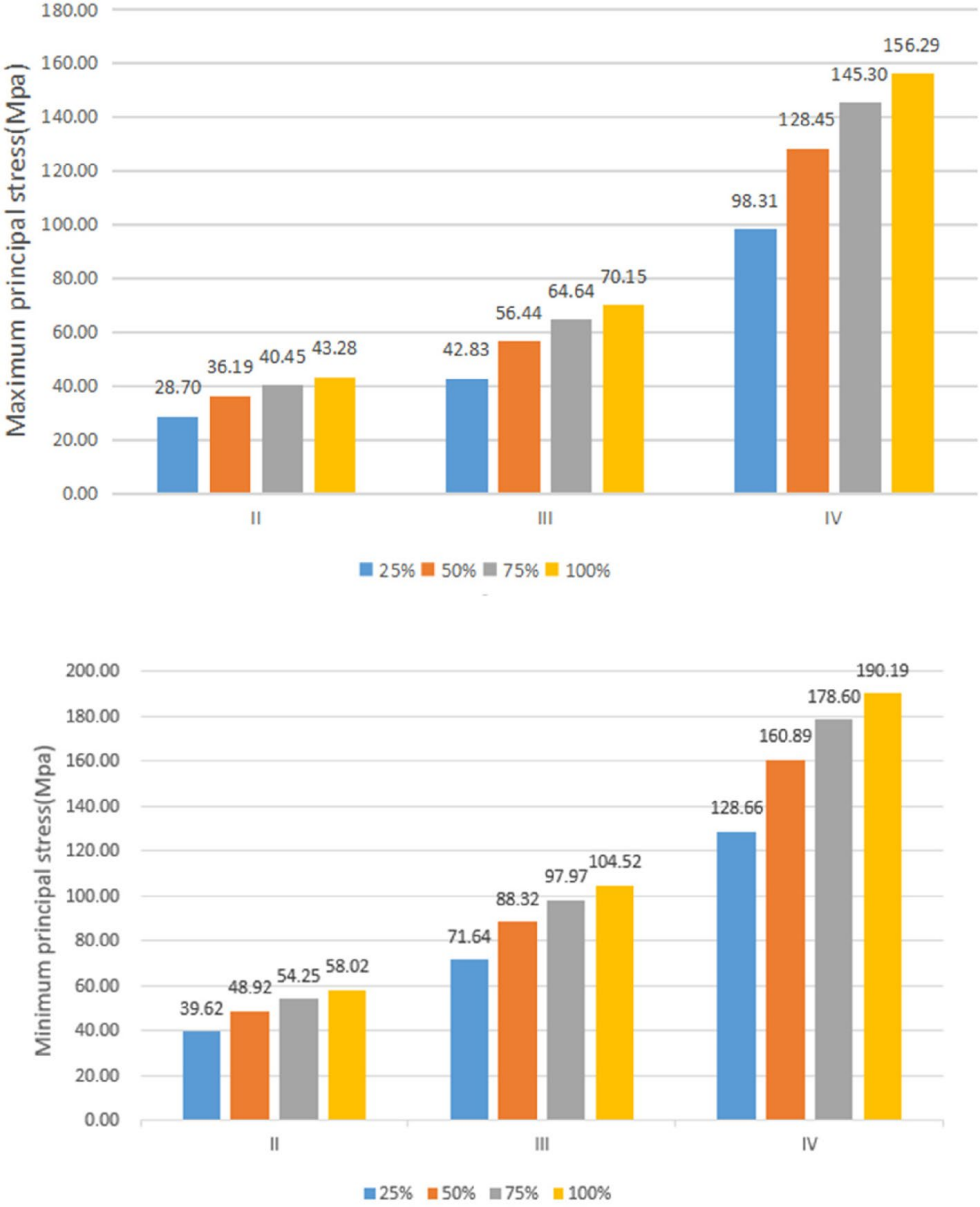
Maximum/minimum principal stresses of cortical bone as a function of the osseointegration rates

**Fig. 12:**
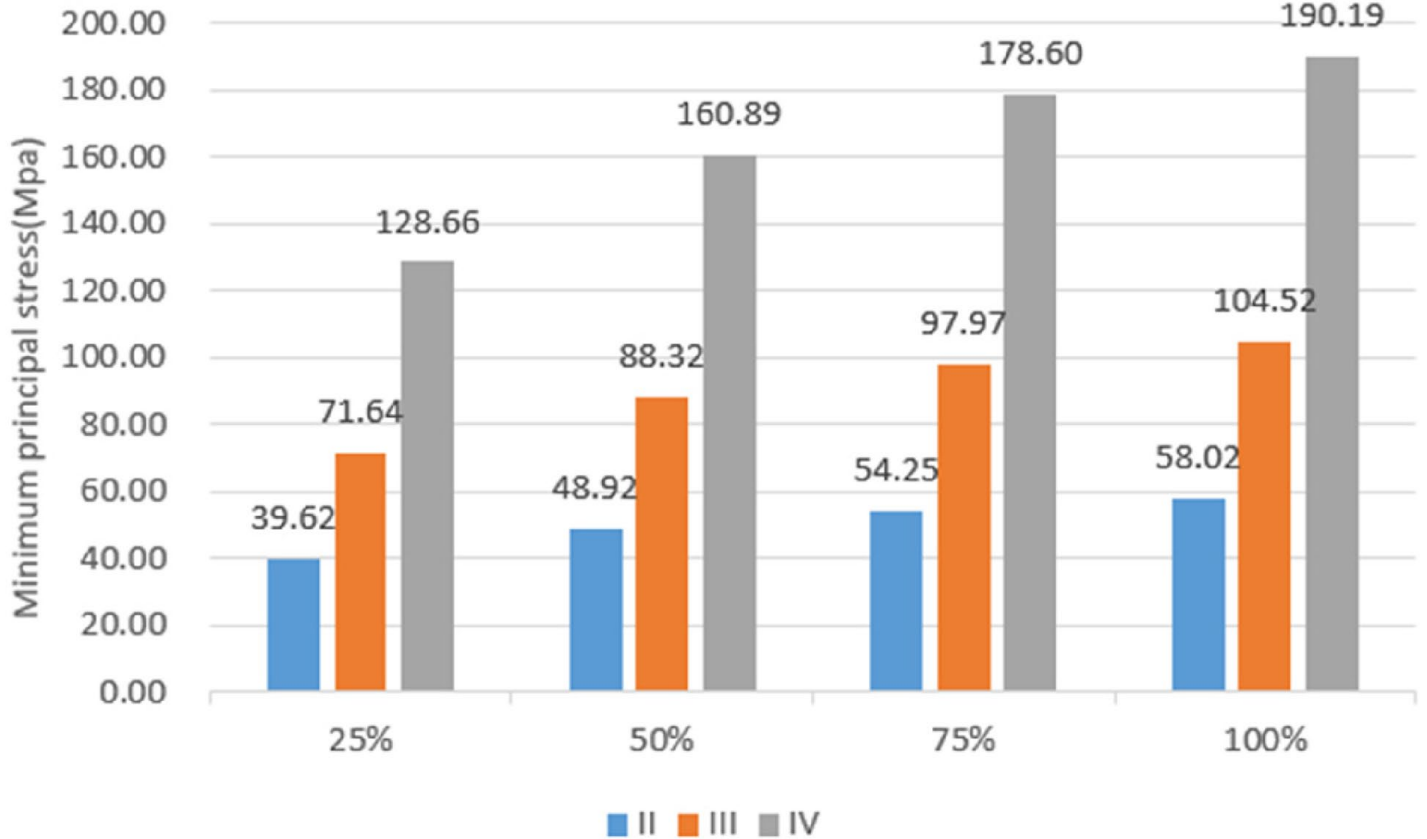
Variation of maximum/minimum principal stresses of cortical bone with bone conditions

Under vertical loading, the outer surface side of cortical bone was the compressed area. The tensile area was the side of cortical bone close to the cancellous bone (Fig. 10a). In the same bone condition, the maximum and minimum principal stresses of cortical bone had increased with increasing osseointegration rates (Fig. 11), but the increment varied with different bone conditions. At a 100% osseointegration rate, the maximum principal stress of cortical bone had increased by 50.8% compared to the maximum principal stress of 25% in Type II, 63.8% in Type III, and 59% in Type IV bone conditions. This indicated that the maximum principal stress of cortical bone in Type III bone was most affected by the osseointegration rate; the minimum principal stress of cortical bone had increased by 46.4% at a 100% osseointegration rate compared with the minimum principal stress at 25% in Type II, 45.95% in Type III, and 47.8% in Type IV bone conditions, which suggested that the effects of osseointegration rates on the minimum principal stress of cortical bone were not significantly different among the three bone conditions. Given the same osseointegration rate, the maximum and minimum principal stresses of cortical bone vary with the bone conditions. The stresses will be greater if the bone conditions are more porotic (Fig. 12).

**Fig. 13:**
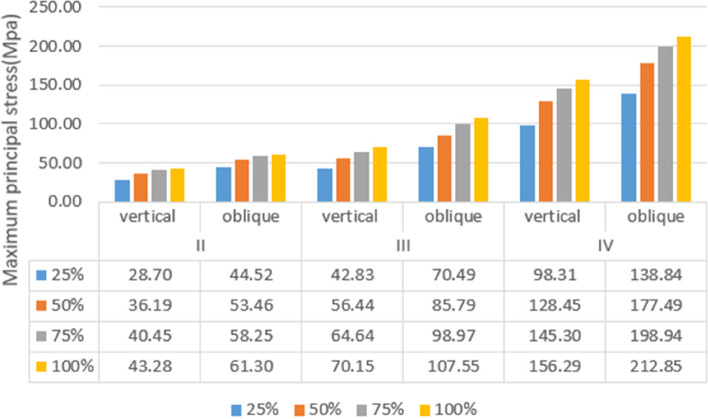
Maximum principal stress of cortical bone under vertical or oblique loading

Under oblique loading, the direction of force application was on the compressed side of cortical bone. The contralateral side was on the tensile side (Fig. 10b). Oblique loading produced more stress than axial loading: 1.3 ~ 1.7 times for tensile stress and 1.4 ~ 1.8 times for compressive stress, respectively (Fig. 13). The stress change during oblique loading was comparable to that during vertical loading. Both tensile and compressive stress increase when the bone condition deteriorates or the osseointegration rate increases. For instance, when the osseointegration rate increases from 25 to 100% in Type II bone conditions, the minimum principal stress increases from 71.5 MPa to 100.9 MPa. The maximum principal stress increases from 44.52 MPa to 61.3 MPa, respectively.

**Fig. 14:**
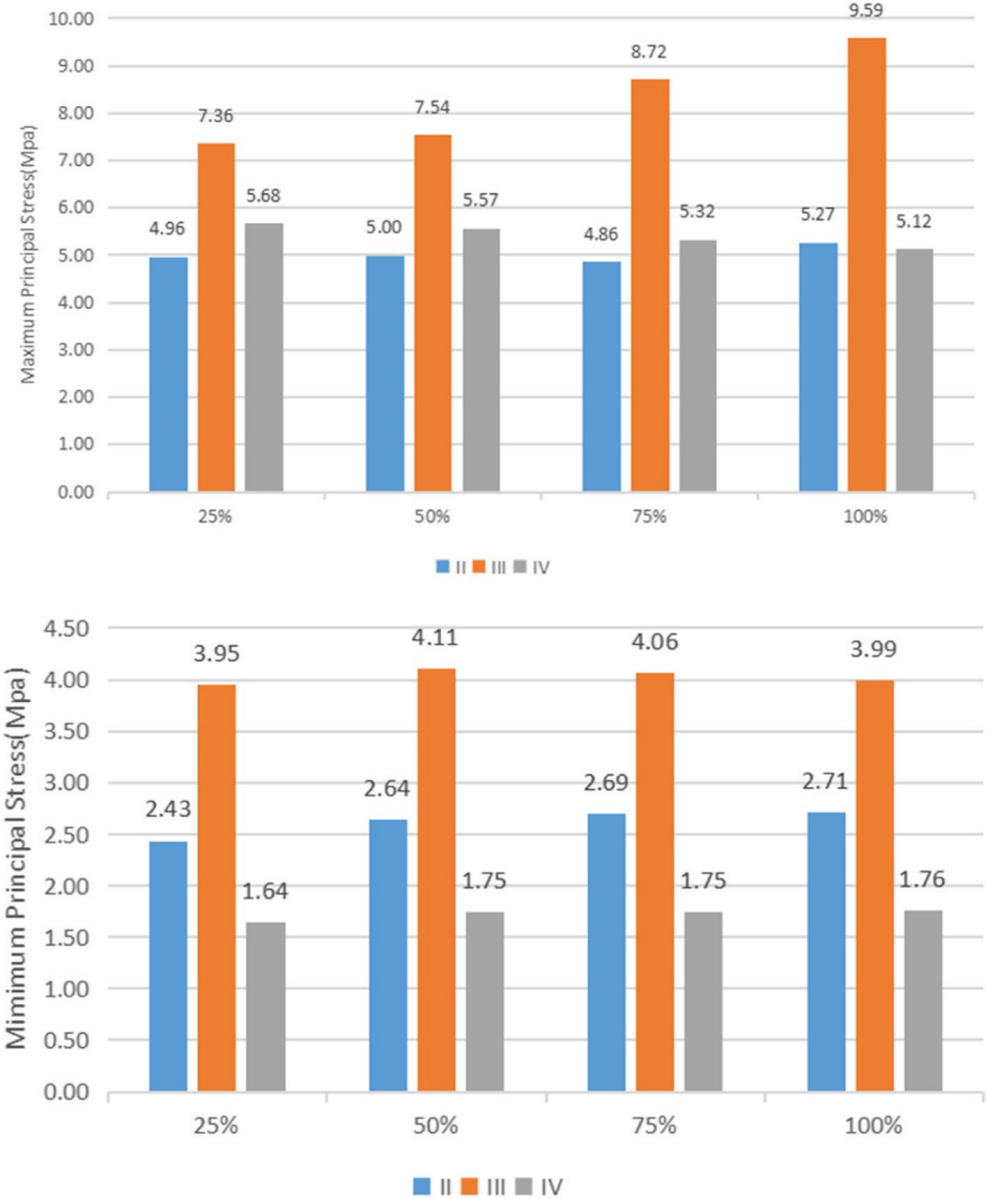
Maximum/minimum principal stresses of cancellous bone

Unlike cortical bone, experimental data suggest that the maximum and minimum principal stresses in cancellous bone always occur in Type III bone regardless of the osseointegration rates and loading directions (axial or oblique) (Fig. 14).

### Maximum shear stress

Table 5 shows the magnitude of shear stresses in the cortical and cancellous bone with different bone conditions, along with the four rates of osseointegration under vertical or oblique loading. Regardless of external conditions, the maximum shear stress occurred in cortical bone and increased with the increasing rates of osseointegration. Shear stress in cancellous bone is much less than that in cortical bone.

**Table 5:** Maximum shear stress values

Bone type		II				III				IV			
BIC		25%	50%	75%	100%	25%	50%	75%	100%	25%	50%	75%	100%
Vertical	Cortical	11.43	12.60	13.13	13.48	18.77	19.51	20.19	20.56	32.75	35.15	36.30	36.79
	Cancellous	1.36	1.45	1.46	1.45	2.76	2.79	2.71	2.63	1.22	1.26	1.23	1.19
Oblique	Cortical	20.87	22.89	24.17	25.96	29.26	31.59	32.75	34.24	47.14	50.21	51.31	51.76
	Cancellous	1.85	1.93	1.93	1.91	3.85	3.96	3.87	3.76	1.57	1.61	1.57	1.53

## Discussion

The crucial aspect for implant osseointegration and its long-term survival is excellent bone quality. Prior studies have demonstrated that the density and quality of cortical and cancellous bone differ based on various factors, such as the patient's age, gender, and health status, as well as the surgical site [20]. The experimental findings indicate that, irrespective of bone conditions, osseointegration rates, or loading directions (i.e., vertical or oblique), the highest stress experienced by bone tissue is at the junction of the implant and cortical bone. Conversely, the stress distribution in cancellous bone is uniform and minimal. This is attributed to the substantially higher elastic modulus of cortical bone compared to cancellous bone, and the resulting stress shielding effect that causes the concentration of stress in the cortical bone [21, 22]. The magnitude of stress in the cortical bone and implant in this study was Type IV > Type III > Type II, indicating that bone with a higher density is better able to distribute loads, whereas bone with a lower density is more prone to implant failure due to overload. The magnitude of stress in cancellous bone is given as Type III > Type IV > Type II, which is because Type III cancellous bone has a larger elastic modulus and requires more force than Type IV cancellous bone.

Enhancing the rate of osseointegration has remained a significant area of interest for researchers in oral implantology. Many 3D finite element studies presume 100% osseointegration which can lead to erroneous results. The lack of differentiation in osseointegration rates is possibly due to the constraints of the FEA modeling software. This study addresses this issue by modeling four osseointegration rates via the assignment of varied properties to specific regions of the implant-bone connection. In this experiment, given the same bone condition, the maximum and minimum principal stresses of cortical bone increased with increased osseointegration rates. The maximum stress was observed in the scenario of 100% osseointegration which is an expected outcome because of the high modulus of elasticity at the implant-bone interface. Conversely, the peak strain in cancellous bone declines as osseointegration increases, implying that superior osseointegration results in less deformation of cancellous bone and enhanced stability of the implant within the bone. This was consistent with the study made by winter et al., who found that the jaws necessarily withstood higher stress and formed lower strains during high osseointegration rates since the elastic modulus of jaws increases and leads to decreased micromotion during the case of high osseointegration rates [23]. In contrast, bones with lower osseointegration rates must compensate for loads through deformation. A stress–strain relationship is a form of energy whereby bone cells expend more energy for bones with lower osseointegration rates, meaning implant failure is more likely to occur at lower osseointegration rates. For implants, however, the strain is relatively small because its elastic modulus is much larger than that of jaw bones, but unlike jaw bones, the strain of implants increases with the increase in osseointegration rates. This phenomenon may be because the strain of the implants is mainly distributed at its junction with cortical bone in the transition region. When the osseointegration rate increases, the increase in elastic modulus of cortical bone is much greater than that of the cancellous bone, and the change in elastic modulus at the interface of cortical bone and cancellous bone increases; thus, the strain of the implants increases at the interface.

Three stresses of concern arise at the bone-implant interface, which are compressive, tensile, and shear stresses. It has been demonstrated that compressive stress has the most favorable effect on bone tissue over a certain range as it increases bone density over time, thereby increasing bone strength. Tensile and shear stresses weaken bones, with shear force being the most damaging [24]. It is evident that when assessing the optimal implant conditions, it is crucial to strike a balance between compressive and tensile stresses while minimizing shear stresses. Achieving this goal may require the design of diverse implant thread forms, an area that warrants future investigation.

To evaluate the implant's safety, it is recommended to first determine whether the stress in the peri-implant bone exceeds the cortical bone ultimate stress. In this study, the maximum Von Mises stress in cortical bone was compared to the cortical bone stress threshold (160 MPa) to evaluate the safety of the cortical bone [25]. In this study, since the bone properties for different osseointegration rates are based on fractional values of bulk bone properties, their corresponding yield strengths must be reduced when osseointegration rates decrease. According to O’Mahony et al., the stress values obtained in this study would increase by 20% to 30% when anisotropic jaw models are applied, when compared to those analyzed by the isotropic models; thus, we believe that the magnitude of stress obtained in this study is large and close to the actual situation compared to similar studies [18]. Based on the experimental findings, using short implants measuring 4.1 mm × 6 mm proved safer under vertical loading when osseointegration rates were ≥ 25% for type II bone, ≥ 50% for type III bone, and 100% for type IV bone. Under inclined loading, the stress did not surpass the threshold for Type II bone with an osseointegration rate of ≥ 50% and Type III bone with an osseointegration rate of 100%. Nevertheless, for Type IV bone, the stress level exceeded the threshold in all loading conditions (i.e., vertical or inclined) across the four osseointegration rates. These outcomes underscore the need for clinicians to preoperatively assess the bone condition at the patient's jaw implant site. If the bone condition is deemed inadequate, clinicians should consider bone grafting or extrusion and weigh the safety of using a short implant. Regarding the implant, the maximum Von Mises stress was concentrated in the neck region where the implant met the cortical bone. The highest stress levels increased with declining bone condition and rising osseointegration rates, but they did not exceed the yield strength of the titanium implant. Consequently, the risk of fatigue fracture of the implant remained low [26].

The maximum stress in implants and cortical bone during oblique loading is higher than that observed during vertical loading, which aligns with the conclusions drawn by other researchers [27, 28]. In this study, the maximum stresses exerted on the implant and cortical bone during inclined loading were higher than those observed during vertical loading. These results suggest that inclined loading is an unfavorable condition for short implants and should be minimized during clinical applications. To ensure implant protection during restorations, implant-protected occlusion design is recommended. This design aims to direct occlusal forces along the long axis of the implant and minimize lateral forces. Restorative designs that can achieve this include: reducing the cusp slope of the restoration and the bucco-lingual and proximal–distal diameters of the restoration; designing a flatter and wider central fossa to allow lateral cusp movement within 1.5 mm of the central fossa during centric occlusion without any bevel obstruction; avoiding occlusal contact during anterior extension and lateral movement to reduce lateral forces during lateral occlusion, and designing reasonable occlusal contact points.

Although the finite element method is an effective way of solving biomechanical problems, there are still limitations in the analytical process of this study. The loads used in this study were within the normal masticatory range of humans, but the loading time and periodicity, distribution, and direction were not adequately taken into account because mastication in the oral cavity is a complicated process, and simulation of the actual masticatory situation requires further research [29]. In this experiment, the superstructure of the implant models was simplified, and the occlusal force was loaded at the center of the abutment surface. However, this showed little effect on the results since the stress distribution law of the implant and its surrounding bone was mainly observed. Unlike in vivo experiments or laboratory experiments, finite element analysis can help calculate and predict the mechanical properties of implant systems. Nevertheless, the oral cavity consists of a complex environment, and its related biochemical behavior needs to be further studied by other means.

## Conclusions

Under these experimental conditions, the following conclusions were drawn from the three-dimensional finite element analysis results:

1. The maximum von mises stresses on jaw bones were concentrated in the cortical bone at the implant neck for all models.
2. As the bone condition deteriorated, the maximum stress of the mandible and implant became larger.
3. Oblique loading is an unfavorable loading condition in which the stress on the mandible and implant is greater than under vertical loading.
4. With the increase in osseointegration rate, the maximum stresses in the mandible and implant gradually increased. Using short implants measuring 4.1 mm × 6 mm was deemed safer under vertical loading conditions when osseointegration rates were ≥ 25% for type II bone, ≥ 50% for type III bone, and 100% for type IV bone. Conversely, under inclined loading, the stress levels did not exceed the threshold for Type II bone with an osseointegration rate of ≥ 50% and Type III bone with an osseointegration rate of 100%. However, stress levels always exceeded the threshold for Type IV bone, regardless of the loading conditions (i.e., vertical or inclined) across the four osseointegration rates.
5. Therefore, when the bone condition is good, and the osseointegration ratio is relatively high, 6 mm short implants can be used.

## Data Availability

The data presented in this study are available on request from the corresponding author.
